# COVID-19 and lockdown impact on BPD patients and their familiars

**DOI:** 10.1192/j.eurpsy.2021.715

**Published:** 2021-08-13

**Authors:** M. Roca Santos, A. Vilaregut Puigdesens, N. Calvo Piñero, T. Pretel Luque, E. Castell Panisello, Z. Nieto Fernandez, M. Ferrer Vinardell

**Affiliations:** 1 Facultat De Psicologia, Ciències De L’educació I L’esport Blanquerna, Universitat Ramon Llull, Barcelona, Spain; 2 Servicio De Psiquiatría, Hospital Universitario Vall d’Hebron, Barcelona, Spain

**Keywords:** Borderline personality disorder, lockdown, family, coronavirus

## Abstract

**Introduction:**

It is large known that Coronavirus outbreak has had a psychological impact on the general population, specifically on those with a mental disease as Borderline Personality Disorder (BPD) and their relatives.

**Objectives:**

The aim of the study is to identify and examine the individual and familiar impact of the coronavirus outbreak on patients diagnosed with BPD and their parents.

**Methods:**

A qualitative research design using focus groups was selected to identify and discuss participants’ experiences, beliefs, perceptions and attitudes. The target population consisted of patients with BPD and their parents. Participants were recruited from the BPD psychiatric service from the Hospital Universitari de la Vall de Hebron (Barcelona, Spain). Data was collected via two focus groups, one with patients with BPD and other with their parents. Content analysis was used to determine categories and themes.

**Results:**

The qualitative analysis of participants’ perceptions are presented using the following themes: changes and difficulties during lockdown, after lockdown concerns and challenges, general learning, and future needs. Results identify factors associated with the COVID-19 outbreak and other factors already present as family dynamics and individual difficulties.

**Conclusions:**

Findings have been discussed focusing on individual and familiar impact, and allows us to consider challenges precipitated by the COVID-19 pandemic. The study evidence that a family intervention approach is essential to enhance BPD treatment.

